# Microscopic characterization of Pseudomonas Aeruginosa confines separate from clinical cases by testing RAPD-PCR method

**Published:** 2015

**Authors:** Z Mahmmudi, A Emami, AA Gorzin

**Affiliations:** * Kazeroun Branch, Islamic Azad University, Kazeroon, Iran; ** Department of Microbiology, Shiraz Burn Research Center, Shiraz University of Medical Sciences, Shiraz, Iran; *** Bacteriology and Virology Department, School of Medicine, Shiraz University of Medical Sciences, Shiraz, Iran

**Keywords:** P. Aeruginosa, RAPD-PCR, genotyping, epidemiology, burn patients

## Abstract

Pseudomonas Aeruginosa is one of the leading opportunistic infectious agents especially in immune-suppressed patients such like burn victims. Carbapenems like Imipenem (IMP) and Meropenem (MEM) are the choice antimicrobial drugs, which used in the treatment of Penicillin and Aminoglycoside-resistant Pseudomonas Aeruginosa isolates. Based on the importance of the detection of infectious source and their resistance transfer algorithm have a main effect on the control of nosocomial infections this study designed for to consider the antibiotic sensitivity and the genetic pattern of Pseudomonas Aeruginosa isolate in burned patients hospitalized in Ghotbeddin Shirazi Hospital with RAPD-PCR technique. According to the Antibiogram results, the most sensitivity was related to the Gentamicin with 50% while the most resistant related to the Nalidixic Acid, Erythromycin, and Cefotaxime with 90% resistant to all. With RAPD-PCR technique used primers 272, 277 and 287 were detected 18, 15, and 11 algorithms respectively. RAPD-PCR is a genotyping method with a high efficiency and good for the study of bacterial epidemiology and polymorphism.

## Introduction

Pseudomonas Aeruginosa is considered one of the most common nosocomial pathogens often causing significant problems in intensive care units [**1**-**4**]. It is common Those P. Aeruginosa diseases linked with notable morbidity and destruction because of the body’s ability to adjust swiftly to variations in the conditions, to quickly produce immunity to medicines, and to provide a diversity of virulence parts [**5**,**29**]. Multidrug-Resistant Bacteria are usually listed as a reason for nosocomial diseases [**6**]. Molecular epidemiologic investigations have an essential function in preparing the transmission paths of a pathogen [**7**]. Some molecular investigations have been performed to examine difference amongst P. Aeruginosa divides, polymorphism of several of its own genes, and including a genetic identification of P. Aeruginosa isolates from various organisms and circumstances [**8**-**11**]. Those were constructed to produce information that would be helpful in developing the common control of infections because of P. Aeruginosa [**14**]. Maximum of these investigations described a special ratio of polymorphism and genetic heterogeneity between P. Aeruginosa separates [**32**,**33**]. Several studies have directed at P. Aeruginosa; most studies confined to the epidemiology and infections, concern outbreaks in patients [**20**]. Only a few articles are present in the endemic state on the genetic investigation of P. Aeruginosa separates from various diseases. Therefore, this study aimed to investigate the antimicrobial sensitivity profile, investigation, and application of RAPD-PCR as an easy molecular technique to detect polymorphism at DNA level among of P. Aeruginosa isolated. Also, determine DNA fingerprinting, genetic distance and phylogenetic diversity of P. Aeruginosa isolated from different sources of infections wounds, ear, burns, urine, sputum, from patients that hospitalized in Ghotbeddin Shirazi Hospital.

## Materials and methods

**Patients **

Over a period of 12 months, two hundred patients from 4 different health centers who diagnosed as having burn diseases studied. The age range of the cases is between 18 months until 85 years. Diagnosis based on the results of the sweat tested the clinical symptoms recorded for each patient [**30**].

**Isolation of P. Aeruginosa**

Wound swabs collected from burn-patients depending on the patient’s age and plated on blood agar plates. Colony morphology reported as non-mucoid or mucoid, and the isolated colonies constrained to official biochemical analyses for the association of P. Aeruginosa. Gram-negative, non-lactose fermenting, oxidase-positive groups that oxidized glucose and set on cetrimide agar in 42ºC identified as P. Aeruginosa. Pigment formation detected on Pseudomonas agar (P-Agar). All isolates kept at –20ºC in media containing 8% dimethyl sulfoxide (DMSO) until use [**22**].

**Antibiotic susceptibility, disk diffusion**

The sensitivity of the P. Aeruginosa isolates determined to Amikacin (AN, 30), Ceftazidime (CAZ, 30), Ciprofloxacin (CP, 5), Cefotaxime (CTX, 30), Gentamicin (GM, 10), Imipenem (IPM, 10), Meropenem (MEM, 10), Chloramphenicol (CK, 30), Erythromycin (E, 10), Nalidixic acid (NA, 30), Piperacillin (PIP, 100 µg), on Mueller-Hinton agar using the disk diffusion assay [12]. The antibiotic disks obtained from two different companies (Mast, England and Pasteur Institute, France). Pseudomonas Aeruginosa ATCC 27853 is used as the laboratory standard for these tests.

**Genomic DNA Isolation**

Total-genomic DNA concluded from fifty isolates having different sites (wounds, burns, urine) using a method described by [**12**]. A particular group treated on 5ml of brain attack strain broth and produced overnight in 36.5ºC. Next, 1.5 ml of a full address was collected by centrifugation for 300sec at 14,000rpm. The cell pellet lysed and resuspended in 200µl of lysis barrier via dynamic pipetting. To eliminate most proteins and cell ruins, 66µl of 5M NaCl resolution combined and stirred fine, and when the viscous compound centrifuged in 11,900rpm for 600sec at 4C. Following selecting the pure supernatant to a novel Eppendorf device, an equivalent amount of chloroform combined, and the device is lightly modified at limited 50 periods during a milky resolution completely made. According to centrifugation 14,000rpm for 300sec., the supernatant later moved to another Eppendorf device and double volume of 00% ethanol combined. The devices inverted 5 to 6 terms lightly, next centrifuged at 10,000rpm for 300 seconds. The supernatant discharged, and 1ml of ethanol (70%) combined to the pellet, and device centrifuged at 10,000rpm for 300sec. Subsequently, the supernatant discharged and the pellet air-dried for 600sec at ambient condition, the pellet resuspended via 100µl H2O. The property kept at -20ºC until management. The DNA collection defined via estimating the absorbance of the specimen at 260nm applying spectrophotometer [**13**].

**RAPD-PCR Amplification**

Three primers employed in this study, these primers were given polymorphisms as listed in (**Table 1**).

**Table 1:** Primers and their sequences utilized in this study

No	Operon code	Nucleotide sequence (5’-3’)
1	272	AGCGGGCCAA
2	277	AGGAAGGTGC
3	287	CGAACGGCGG

Amplifications with each primer were performed in 50µl consist of 5µm buffer 10x reaction with MgCl2 (Taq plus), 1.2µm dNTPs 10 (Mm), 2µm primer ten Pmol, 0.4µm Taq DNA polymerase 5 unit 0.5ng of genomic DNA and 36.4 Distilled Water. The amplification was performed in a programmed thermocycler. Its procedure is as: 1 cycle 95ºC for 5 minutes, 36 cycles (95ºC for 1 minute); {42ºC for 3 minutes for primer 272,38ºC for 3 minutes for primer 277; 44ºC for 3 minutes for primer 287}, 72ºC for 2 minutes) and 1 cycle 72ºC for five minutes. The addition goods determined by electrophoresis on a 1.5% agarose gel in 1X TBE buffer at 70 volts for 3.5 hr (5 volt/ cm) [**16**,**18**]. Gels decorated via Ethidium bromide, visualized in UV light and photographed utilizing a high-resolution digital camera (12.1 megapixels), nominal molecular weight markers furthers employed in per electrophoresis run [**15**,**17**].

**RAPD Data Scoring and Analysis**

The PCR-based DNA marker RAPD used in this study as an accessible tool for analyzing the polymorphism, genetic variation and fingerprinting of P. Aeruginosa isolates collected from different sites of infections [**22**]. RAPD outcomes analyzed by utilizing all information obtained from tables and figures. This information included the presence or absence of amplified DNA bands, the whole amount of boosted bands across all separates of P. Aeruginosa, the number of polymorphic bands, which can be detected horizontally. 

J. of the University of a Bar for pure science: The Mathematical Taxonomy System (NTSYS) 1.8, working the Jaccard factor of identity [**16**], Unweight pair team system arithmetic means group investigation applied to calculate genetic distance and obtaining phylogenetic tree [**23**]. Primer efficiency and discriminatory power calculated for each primer using two equations as described by [**19**,**21**].

## Results

**Results of antibiogram **

**Fig. 1:**
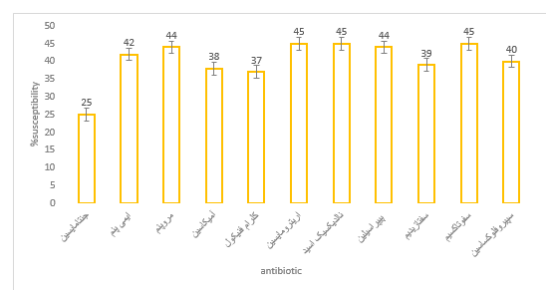
The antibiotic susceptibility of Pseudomonas Aeruginosa isolates from the Iranian Burn patients using the disk diffusion method. Amikacin (AN), Ceftazidime (CAZ), Chloramphenicol (CK), Erythromycin (E), Nalidixic acid (NA), Cefotaxime (CTX), Ciprofloxacin (CP), Gentamicin (GN), Imipenem (IPM), Meropenem (MEM), Piperacillin (PIP), and Piperacillin (PIP) were used

The findings of the antibiotic susceptibility of the separates using the disk method showed in **Fig. 1**. The multi-drug resistant rate was 75%, in which most of them were from Men (56.6%). 20% isolates were sensitive to Imipenem and Meropenem, the two antibiotics that are used to treat P. Aeruginosa infections of burn patients in Iran. Susceptibility to the other antibiotics was; 35%sensitivity to ciprofloxacin, 10% to Ceftazidime, 32% to Amikacin and Piperacillin and Chloramphenicol, 37% to Erythromycin and Nalidixic acid and Cefotaxime, 50% to Gentamicin. 

**Results of RAPD-PCR**

**Fig. 2:**
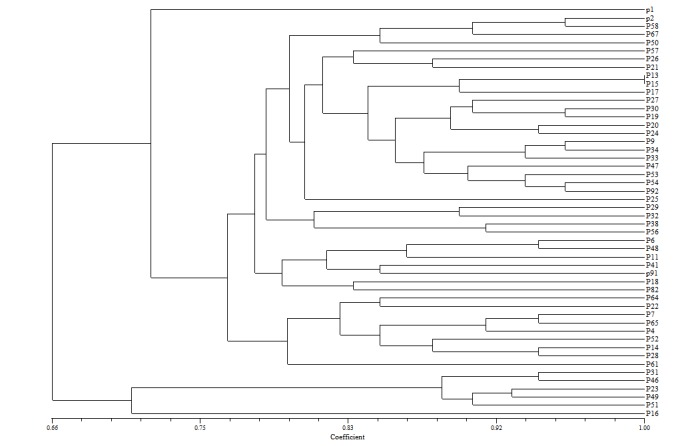
Dendrogram of Pseudomonas Aeruginosa isolates showing percent similarities of patterns

Fifty cases of Pseudomonas Aeruginosa isolated in the microbiology laboratory in Ghotbeddin Shirazi hospital during a one-year period. Twenty eight cases were from patients admitted to ICU, 35 cases from Pediatric, 83 cases from men and 54 cases from women that showed in **Fig. 2**. Using primer 272, 22 diversity of bands (200-2300bp), primer 277, 17 diversity of bands (400-2700bp) primer 287, 15 diversity of bands (300-3000bp), were detected. The resultant dendrogram produced by GelCompar II software (**Fig. 2**) showed 40-100% genetic homology between the isolates. With the 50 isolates studied, the PCR with three primers generated 54 different patterns, including four clones detected mostly from men.

**Table 2:** The amount of bands, efficiency & discriminatory value of each RAPD primer gave amplification product in this study

No	Primer code	Nucleotide sequence 5 to 3	No. of. bands	No. of. polymorphic bands	Primer efficiency	Discriminatory Power %
1	272	AGCGGGCCAA	86	22	45.3	%43.1
2	277	AGAAAGGTGC	65	17	34.2	%33.4
3	287	CGAACGGCGG	39	12	20.5	%23.5
	Total		190	51		

The use of Three RAPD primers produced a whole of 190 boosted bands and 51 polymorphic bands, this shows that there is a clear genetic diversity among the isolates. Based on the amount of revealed bands each primer, they varied among (2 and 7) (**Table 2**). The high number of RAPD patterns was shown in primer 272 (86 bands), while the lowest number was shown with primer 287 (only 39 groups). The high number of bands attributed to the presence of a significant number of primer annealing sites the template DNA of the tested isolates. The largest number of bands usually gives a better chance for detecting polymorphisms among individuals [**22**].

High level of polymorphism was presented with primer 272 (22 groups), while the lower level of polymorphism showed with primer 287 (12 bands) (**Table 2**). The differences in molecular weights of polymorphic bands reflect the number of targets for each primer site locus within the DNA in question [**23**,**24**]. Polymorphisms at DNA level may occur because of several types of mutations, like the specified base variation in the primer-annealing site in the genome that prevents addition by adding a mismatch at 3‘end of a DNA section [**25**]. Other causes of polymorphisms may involve deletion of a priming position, the injection that does priming places to be extremely cold to sustain increase, or they may adjust the quantity of DNA part outdoors limiting its increase [**26**]. The failure of many primers to amplify DNA may be due to their need to special requirements for amplifications regarding PCR-reagents or heat outline because all of the response factors were indistinguishable for all textbooks. Furthermore, variations in banding designs are possible because of particular conditions of a distributed primer. The G+C satisfied of the primer may further interfere with PCR yield [**22**,**27**].

## Discussion 

 The purpose of strain typing studies is to provide laboratory evidence that the epidemiologically associated separates obtained through an explosion of attack are also related genetically and thus represent the same strain. This information is helpful for understanding and controlling the spread of illness in hospital and communities [**12**,**23**]. In this study, we have determined the genetic diversity of Pseudomonas Aeruginosa strains isolated from different departments of Ghotbeddin Shirazi Hospital in an endemic situation, using RAPD analysis. The results compared with those of antibiogram base on NCCLS. In agreement with other studies, there was substantial diversity among the strains. The large numbers of genotypes suggest that most strains Pseudomonas Aeruginosa were derived from the patients themselves, as shown previously [**3**,**5**]. In this study, a few genetically related isolates (4 clones) detected were mostly from men. Nevertheless, the epidemiological results should ever be recorded in record when determining either genetically associated forces are further related epidemiologically. Epidemiologically linked species defined as species cultured from patients’ specimens collected in a limited period or from designated area as a part of the epidemiologic study and these might have a common source [**12**]. However, cross-acquisition was established for only seven patients (3 pairs isolates in three clones: 1, 2, 4), these results are suggestive of a common exogenous source. This only found for clone 4, in which one environmental sample from the ventilator obtained. 

Speijer et al. [**3**], in their study on Pseudomonas isolated from burn patients in endemic conditions, showed that most patients infected during their stay in ICU, which indicated that a common exogenous source or cross-acquisition was an important route of Pseudomonas Aeruginosa transmission. In our study, isolation of clone from burn patients was in accord with their findings. However, in their study, most patients were infected with different species of Pseudomonas; this could be indicative of an endogenous source that did not detect on admission. We also had multiple different genotypes of Pseudomonas Aeruginosa. Computer analysis of banding pattern revealed different groups of genetically related species. Discrepancies were present in results of computer analysis and visual observation, which have been present in other studies as well [**1**]. To identify whether the genetically related species are also epidemiologically related, in addition to the comparison of the eye observation (visual exam) and GelCompar, epidemiologic information should also be included [**5**]. RAPD method recommended as an excellent screening method for many bacterial species; this has had comparable results with the PFGE reference method which is very expensive and time-consuming [**2**,**5**,**13**]. The selection of introductions for use in RAPD analysis is one of the most vita parameters. It appears that some arbitrary primers may work easier than others and may provide results that are more reproducible [**14**]. We used the three sets of primers that were used in previous studies and had comparable results with PFGE method [**4**]. No association observed between genotype and antibiotype as isolates of the same genotype displayed different antibiotype and vice versa, as already shown by others [**15**]. The most effective antibiotic agent in our study was Gentamicin. The incidence of resistance is dependent on the patterns of antibiotic usage and is different in other countries [**16**,**28**]. In one study, performed in Brazil, showed that Imipenem is the most active agent against P. Aeruginosa followed by Ciprofloxacin [**23**]. There was a high antibiotic resistance rate mostly in our Men, in which a multi-drug resistance rate was 75%. Loureiro et al. [**2**] showed 75-100% strength in specimens obtained in Men. The least antibiotic resistance was to Terazosin (0-35%).

## Conclusion

Control of infections is based on the identification of the organisms and their mode of spread; the molecular technique used in this study makes this possible in the shortest possible time with a reasonable cost. Results of this study showed that Antibiotic resistance in Pseudomonas Aeruginosa isolates in this center is in increasing while their genetic pattern is different in various wards. According to that, the pediatrics wards have the same algorithm with other wards it concluded that this ward contaminated with other departments of the hospital. During male and female, wards had their particular genetic pattern. The conclusion from the total results of the study shows that control of infection among different departments of this clinical center is crucial and manly must control with clinicians and staffs who works in various wards.
